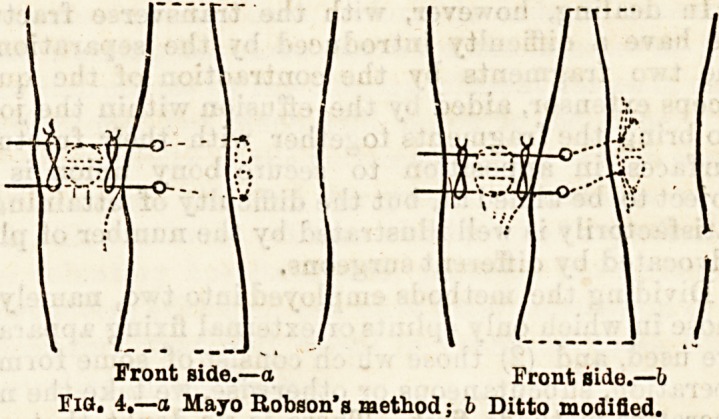# Treatment of Fracture of the Patella

**Published:** 1892-10-01

**Authors:** 


### The Hospital Clinic.

[*The Editor will be glad to receive offers of co-operation and contributions from members of the profession. All letters should be addressed to* The Editor, The Lodge, Porchester Square, London, W.]

### ST. THOMAS'S HOSPITAL.

### Treatment of Fracture of the Patella.

Of the various kinds of simple fracture of the patella the transverse one is by far the most important, and it is of this that the present article mainly treats, as it is the one for which a very great variety of methods of treatment have been recommended and practised by different surgeons, Many of the methods given in the various text-books have fallen already into disuse, and new plans, or modification of older ones, are frequently being introduced, so that it is impossible in the limits of a short article to give a comprehensive sketch of the whole question.

We propose only to describe the methods generally employed at St. Thomas's Hospital.

We may dismiss in a few words the treatment of simple longitudinal or stellate fracture unaccompanied by any serious separation of the fragments. A back splint, such as a MacIntyre, or similar apparatus to keep the limb at rest, and an ice bag, or cool dressing with some evaporating lotion to keep down the inflammation and effusion into the joint (usually somewhat severe, as such injuries are generally the result of direct violence), are all that are required at first, unless the effusion should be so extensive as to make aseptic aspiration of the joint advisable. After about a week the inflammation will have usually subsided sufficiently to allow the limb to be put up in a plaster of Paris splint, which should be kept on for about a month, though the patient can usually get about with crutches after a fortnight.

In dealing, however, with the transverse fracture, we have a difficulty introduced by the separation of the two fragments by the contraction of the quadriceps extensor, aided by the effusion within the joint. To bring the fragments together with their fractured surfaces in apposition to secure bony union is the object to be aimed at, but the difficulty of attaining it satisfactorily is well illustrated by the number of plans advocated by different surgeons.

Dividing the methods employed into two, namely (1) those in which only splints or external fixing apparatus are used, and (2) those which consist of some form of operation, subcutaneous or otherwise, we take the non-operative methods first. There is no doubt that good union and a sound useful limb can be obtained by the careful use of splints. The union may not be bony, and there may be some irregularity or even marked interval between the edges of the fragments as felt through the skin, but it must be remembered that a firm fibrous union is possibly stronger than the bone, for in most cases of re-fracture of the patella it is the bone and not the tough uniting fibre of the old fracture that gives way. The question of time, however, is more important. A fracture treated by splints alone will not stand the strain of use, at any rate use implying flexion, for nine or even twelve months after the injury, for it is liable to stretch slowly until the fragments become widely separated and the stability of the limb is lost, whereas in cases where the fragments have been united by any efficient form of suture, the limb can be used for anything except violent exercise after a month or two. The "pros" and "cons" of operative and non-operative treatment are well discussed in the last edition of Erichsen's Surgery, and to go over them in detail is beyond the scope of the present article. Suffice it to say that where asepsis can be absolutely relied upon operative treatment is more certain and much more rapid in restoring the usefulness of the limb, but if asepsis is doubtful, operation offers a serious risk to the limb and even the life of the patient. The splint treatment, if carefully carried out, should be perfectly efficient, and, though slow, is absolutely safe.

The following are the methods which have generally been employed where operation has not been practised.

If the fracture is seen very soon after the accident, *i.e.*, before much effusion into the point has occurred, it is possible to bring the fragments into apposition by a pad of lint placed above the upper fragment and held in position by a strip of strapping plaister over the pad and passing obliquely down to embrace the calf of the leg. A plaster of Paris splint is then applied to the limb from a little above the ankle to about the middle of the thigh, an oval window being left over the front of the knee joint through which the condition of the patella and joint can be watched, and to which an ice-bag may be applied for a few days to keep down the effusion.

It is not necessary to do more than fix the lower fragment in position by a strip of strapping passing round the limb, as there is no muscular force tending to displace it. The form of plaster of Paris splint employed is that introduced by Mr. Croft, consisting of two side pieces of Bavarian or coarse flannel cut so as to meet accurately down the midline of the limb in front and behind, except over the patella, the "fit" being obtained by careful measurement of the limb at various points, the width of each side piece being half the circumference of the limb at the point where it will lie when applied. The side pieces, each consisting of a double layer of flannel, are saturated with a thick cream of freshly-mixed plaster of Paris in water, and bandaged on over a layer

Fig. 1.—*a* A bag suspended from cradle over limb; *b* Plaster of Paris splint; *c* Pads fixed over fragments by strapping.

of lint or wool before the plaster sets. The pad over the upper fragment may require readjusting occasionally to keep the two fragments in apposition, and as this involves the removal of the plaster of Paris splint, a modification of the above plan has been employed sometimes with good results—namely, the application of a plaster of Paris splint to the limb direct, with a large window over the patella, leaving room for the application of the pad and strapping plaster over the fragments outside the splint.

Another method occasionally employed is the application of a double layer of Bavarian flannel cut into a kind of horseshoe shape, soaked in plaster of Paris as above, and applied anteriorly so as to embrace the front and sides of the limb, the hollow of the horseshoe lying over the upper fragment of the patella, which is held firmly in position while the splint is bandaged on. A similar "horseshoe" is now applied below, embracing the lower fragment. The whole forms one continuous anterior and lateral splint, with a gap over the patella, but united by a bridge at each side, formed of the overlapping "legs" of the horseshoe pieces. Before the plaster sets a finger is thrust down under these "bridges," so that when firm they form arches, and do not press on the side of the limb to interfere with the blood supply of the patella.

Fig. 2.—*a* Upper piece of plaster of Paris splint; *b* Lower ditto; *c* Points where the splint material is raised from the limb before it gets hard, so that arches are made on either side, taking off pressure from the blood vessels to the patella; *d* Point where wool is inserted to keep up pressure on the upper fragment, as the effusion subsides; *e* The bandage near the plaster is omitted in the figure.

In the course of the first ten days, as the effusion subsides, the splint presses less and less firmly on the upper fragment, but this is corrected by tucking in pieces of absorbent wool day after day, following up the retreating effusion, and thus keeping a continuous pressure on the upper fragment of the patella.

In both the above methods the splint must be kept on and the patient kept in bed for six weeks at least. He may then get up with crutches, but should not leave off the splint for several months, and even then he should replace it by a stiff leather apparatus, to be worn for nine months or a year. The stiffness in the joint resulting from long disuse generally disappears gradually with use, and seldom requires more than gentle, passive movement, if any. Should the case not be seen for some time after the accident, so that considerable effusion has taken place, it is best to reduce this before applying the splint, either by resting the limb in a MacIntyre or other back splint, with an ice-bag applied for a few days, or else to aspirate the joint, but the latter method has seldom been employed at St. Thomas's Hospital. These remarks also apply to the first three of the operative methods to be now described.

Of the various operative methods, the following four have been employed by different surgeons at St. Thomas's Hospital:—

(1) Kocker's method, consisting of the introduction of a thick silver wire through the joint, including both fragments, the two ends of the wire being brought together externally over a folded piece of lint or gutta-percha, thus drawing the fragments together (Fig. 3). The wire is passed by a long, slightly curved mounted needle, with all antiseptic precaution, and after the ends of the wire are twisted up, an antiseptic dressing is applied, and the limb laid on a back splint. In three weeks the wire may be removed, and a plaster of Paris splint applied to enclose the limb. This is worn for about two months more, though the patient may walk a little with crutches after six weeks. When the plaster splint is removed the patient should wear a leather support-

Fig. 3.—Kocker's method.

ing apparatus for from three to six months more, according to the security of the union obtained.

(2) Mayo Robson's method, in which two long steel pins are passed, one through the tendon just above the upper fragment, and the other through the ligamentum patellæ just below the lower fragment. These are then drawn together by silk or rubber bands, so as to bring the fragments in apposition. An antiseptic dressing is applied over these, and the limb laid on a back splint. The rest of the treatment is exactly the same as in No. 1.

(3) A modification of the above, in which the pins are passed through the fibrous tissues lying over the surface of the patellar fragments, it being claimed that better apposition of the fractured surfaces is obtained, as there is less tendency to the reversion of the fragments often observed where No. 2 method is employed, the rest of the details of treatment being the same as in No. 2.

Front side.—*a* Front side.—*b*

Fig. 4.—*a* Mayo Robson's method; *b* Ditto modified.

(4) The open method, in which an incision is made across the joint, the blood clot and synovial effusion washed out with antiseptic solution (usually hydrarg. perchlor., strength about 1 in 4,000), the raw surfaces cleaned of any inverted fibres of tissue from the torn ligaments, and the fragments united by silver wire sutures passing obliquely through from the anterior surface to the raw or fractured surface near the articular cartilage. The fragments are brought together accurately and the wires twisted and cut off short, the ends gently hammered down on to the bone to get rid of rough points. The skin wound is then closed after allowing for drainage, either at the side or by a special opening made posteriorly. An antiseptic dressing and a back splint or plaster of Paris "case" is applied, and, except when interference is necessary on account of attention to the drainage, or removal of superficial sutures, the joint may be left untouched for three weeks, after which the patient can usually be allowed to get up with the splint on, and in about six weeks or two months can use the limb, if all has gone well, for anything short of violent exercise, no splint being necessary.

It should be understood that for such an admirable result the most perfect asepsis is necessary, and the most accurate apposition of the fragments. We know of one case at another hospital where after an open operation a perfect result seemed to have been obtained, until, a few months after the operation, the fragments separated suddenly when the patient was going upstairs. On examination it was found that the bones had never united, but had been held together by the wire alone. The re-fracture, therefore, took place in the wire. We may conclude our remarks by saying that the "open method" is, obviously, the only one applicable to compound fractures, in which extra care must be taken in washing out the joint, owing to the possibility of the presence of foreign matter; and given successful antiseptic measures, these cases do as well as any.

## Figures and Tables

**Fig. 1. f1:**
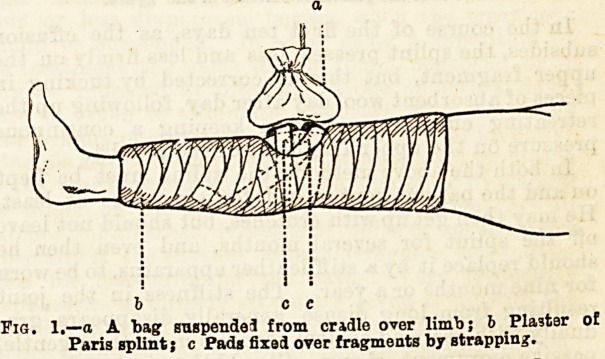


**Fig. 2. f2:**
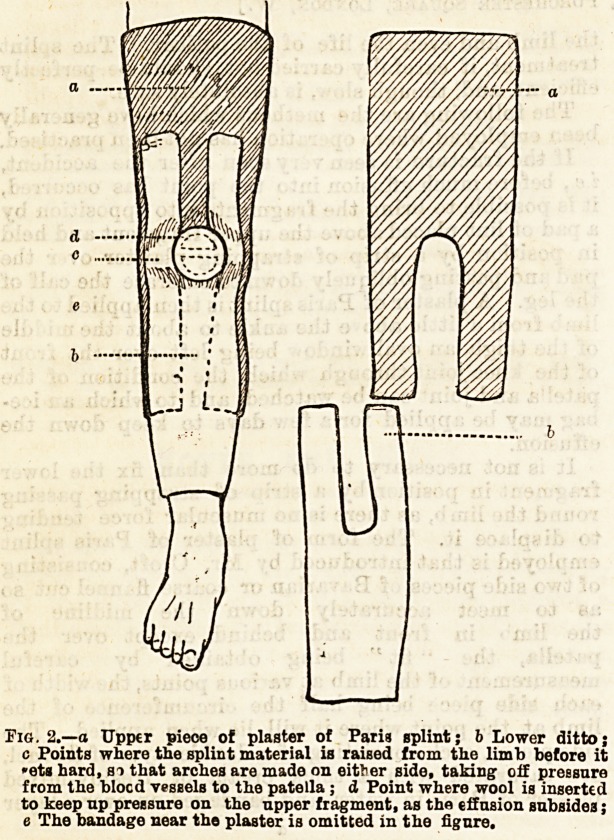


**Fig. 3. f3:**
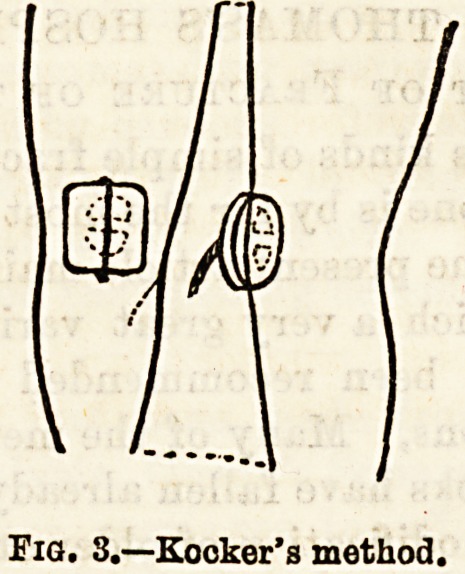


**Fig. 4. f4:**